# Evaluation of the effect of trans sodium crocetinate and crocetin on reperfusion injury in acute myocardial infarction with ST-segment elevation: A double-blind, randomized, placebo-controlled clinical trial

**DOI:** 10.22038/ajp.2025.26046

**Published:** 2026

**Authors:** Ghazaleh Elahabadi, Arash Gholoobi, Javad Ramezani, Ali Eshraghi, Reza Javidi Dasht Bayaz, Vahid Ghavami, Majid Sezavar Dokht faroughi, Amir Hooshang Mohammadpour, Hossein Hosseinzadeh

**Affiliations:** 1 *Department of Clinical Pharmacy, School of Pharmacy, Mashhad University of Medical Sciences, Mashhad, Iran *; 2 *Department of Cardiovascular Diseases, Faculty of Medicine, Mashhad University of Medical Sciences, Mashhad, Iran*; 3 *Department of Biostatistics, Social Determinants of Health Research Center, Mashhad University of Medical Sciences, Mashhad, Iran*; 4 *Traditional Department of Pediatrics, Faculty of Medicine, Mashhad University of Medical Sciences, Mashhad, Iran*; 5 *Pharmaceutical Research Center, Pharmaceutical Technology Institute, Mashhad University of Medical Sciences, Mashhad, Iran*; 6 *Department of Pharmacodynamics and Toxicology, School of Pharmacy, Mashhad University of Medical Sciences, Mashhad, Iran*

**Keywords:** Trans sodium crocetinate Crocetin, Reperfusion injury, ST-elevation myocardial infarction

## Abstract

**Objective:**

This randomized, double-blind trial evaluated trans sodium crocetinate (TSC)—a crocetin-derived antioxidant and crocetin with potential cardioprotective effects—on reperfusion injury in 90 ST-elevation myocardial infarction (STEMI) patients after primary percutaneous coronary intervention (PPCI).

**Materials and Methods:**

Patients received either TSC (0.5 mg/kg injection pre-PPCI + 7.5 mg crocetin tablets for 3 days) or placebo. The primary outcome was ≥ 70% ST-segment resolution 1-hr post-PPCI. Secondary outcomes included corrected thrombolysis in myocardial infarction frame count (CTIMIFC), arrhythmia rates, and echocardiographic parameters (left ventricular ejection fraction (LVEF) and LV size).

**Results:**

ST-segment resolution occurrence was significantly higher in the TSC group versus placebo (p=0.018). There was no difference in CTIMIFC between the two groups. Echocardiographic parameters were similar between the TSC and placebo groups. Although not statistically significant, the frequency of supraventricular and ventricular arrhythmias was lower in the TSC group. Adverse drug effects were comparable between the two groups.

**Conclusion:**

TSC (0.5 mg/kg injection pre-PPCI + 7.5 mg crocetin tablets for 3 days) administration improved myocardial reperfusion, as evidenced by enhanced ST-segment resolution, suggesting reduced reperfusion injury in STEMI patients post-PPCI. While no benefits were observed in CTIMIFC or cardiac remodeling, the safety profile and primary outcome results support further investigation. Larger trials are needed to confirm efficacy and assess long-term clinical impacts.

## Introduction

Acute myocardial infarction (AMI) is certainly among the most serious and dangerous complications of coronary artery disease (CAD) globally (Anderson and Morrow 2017, Hausenloy, Botker et al. 2017, Reed, Rossi et al. 2017). ST-segment elevation myocardial infarction (STEMI) is considered the most critical and serious type of CAD, and it is frequently linked to considerable morbidity and mortality (Choudhury, West et al. 2016, Vogel, Claessen et al. 2019). Primary percutaneous coronary intervention (PPCI) is an essential invasive technique employed to treat AMI and has the potential to enhance clinical results (Hausenloy and Yellon 2008, Khan and Ludman 2022). Primary PCI can cause myocardial reperfusion injury (MRI) due to the reperfusion process during myocardial infarction. This can lead to cardiomyocyte death (Fröhlich, Meier et al. 2013). This process itself can paradoxically cause injury to the myocardium (Yellon and Hausenloy 2007) such as myocardial stunning, ventricular arrhythmias, and microvascular dysfunction (Piot, Croisille et al. 2008). This injury can significantly affect clinical outcomes, even after successful reperfusion therapies such as PCI (Yellon and Hausenloy 2007). Reperfusion following a myocardial infarction can lead to myocardial damage and worsen clinical results, potentially contributing as much as 50% to the final size of the infarct (Fröhlich, Meier et al. 2013, Xiang, Lu et al. 2021). The pathophysiology and pathogenesis of myocardial ischemia-reperfusion injury involve various mechanisms such as oxidative stress, apoptosis, autophagy, inflammation, mitochondrial Permeability Transition Pore dysfunction, changes in intracellular calcium homeostasis, altered myocardial metabolism, production of free fatty acids and immune responses (Verma, Fedak et al. 2002, Yellon and Hausenloy 2007, Bainey and Armstrong 2014, Wang, Li et al. 2021, Xiang, Lu et al. 2021). Oxidative stress is a key mechanism of reperfusion injury, which can lead to the accumulation of reactive oxygen species (ROS) and exacerbation of cellular damage (Xiang, Lu et al. 2021). Excess production of ROS remains a focal point in understanding the mechanisms of reperfusion injury (Marczin, El-Habashi et al. 2003, Granger and Kvietys 2015, Zhou, Chuang et al. 2015). The impairment in blood flow and endothelial dysfunction results from ion channel alterations and elevated oxidative stress. This process is believed to be facilitated by ROS, considered the main causal factor of myocardial injury during reperfusion (Zhang, Brennan et al. 2002, Matin, Ghaffari et al. 2020).

Saffron contains over 150 chemicals; crocin and crocetin are the most biologically active compounds (Hosseini, Razavi et al. 2018). Crocetin is a compound classified as an apocarotenoid carboxylic acid. It does pose challenges in terms of its solubility (Guo, Li et al. 2022, Klunko, Achmad et al. 2023). Crocetin is an antioxidant with other pharmacological effects (Ajzashokouhi, Razavi et al. 2024). Crocin converts to crocetin in the intestines but with low plasma levels intravenously (Hosseini, Razavi et al. 2018). Trans sodium crocetinate (TSC) is a compound derived from crocetin. TSC has displayed the potential to enhance the diffusion of small molecules in aqueous solutions by altering the structure of water molecules (Chang, Chen et al. 2019, Streinu-Cercel, Săndulescu et al. 2021). The findings from the Chang et al study uncovered a profound cardioprotective effect of TSC in the context of myocardial infarction (MI) and reperfusion injury. Their findings demonstrate that TSC reduces oxidative stress, apoptosis, and mitochondrial dysfunction in the heart tissue caused by MI and reperfusion injury through the mediation of the SIRT3/FOXO3a/SOD2 signaling pathway (Chang, Chen et al. 2019).

Considering the absence of effective pharmacological treatments for post-MRI in reperfused STEMI patients and the significance of oxidative stress as a key contributor to reperfusion injury, alongside animal research suggesting that TSC can lessen oxidative stress, we initiated the first human trial to assess the impacts of TSC on reperfusion injury in STEMI. 

## Materials and Methods

### Study design

The prospective randomized, double-blind, placebo-control study was conducted at Imam Reza Hospital, Mashhad, Iran between June 2022 to September 2023. The study was registered at the Iranian Registry of Clinical Trials (registration number IRCT20120520009801N5) and approved by the Internal Review Board and Ethics Committee of Mashhad University of Medical Sciences (IR.MUMS.REC.1400.131).

### Patient selection

Every patient was notified and provided their written consent. The criteria for inclusion were outlined as follows: 1) Ages between 18 to 78, 2) Present of ST elevation on ECG with the clinical decision to treat with PPCI, 3) Thrombolysis In Myocardial Infarction(TIMI )flow zero, 4) The onset of pain is between 0 and 6 hr, 5) No prior hypersensitivity to saffron or its component, and 6) Not pregnant women.

Exclusion criteria were as follows: 1) cardiac arrest, 2) ventricular fibrillation, 3) cardiogenic shock, 4) history of previous AMI, 5) liver failure Alanine aminotransferase AST/aspartate aminotransferase ALT>5Upper limit normal ULN, 6) having Intra-aortic balloon pump, 7) Glomerular filtration rate GFR less than 60 ml/min, 8) history of chronic inflammatory disease, or 8) Left main coronary artery LMCA involvement. Patients who declined to participate did not enter the study.

### Randomization and blinding

Randomization followed the block method utilizing uniform block sizes. A random grouping of two sets consisted of treatment (T) and control (C) across 23 blocks using www.sealedenvelope.com for 92 participants. Randomization in blocks is explained as having 4 members in each block, shaped as follows: [AABB], [ABAB], [ABBA], [BABA], [BBAA], [BAAB]. The vials and pillboxes were labeled as A and B for distinction. To conceal the random assignment, vials and boxes of identical shape were utilized for both the drug and placebo, and these boxes were matched to the random order of A and B with the drug or placebo; the row numbers 1 to 92 and the ID code were noted on them. As the placebo was produced by DR-Rajabi Pharmaceutical company, just like the TSC vials and crocetin tablets, we had no problem in creating masks, since both the drug and placebo had identical color and shape. The preparation and organization of the random sequence are solely under the control of the study’s epidemiologist. Upon entering the study, each candidate was given the vials and pill boxes that corresponded to their unique number. All study participants, such as patients, doctors, nurses, and clinical pharmacy residents, did not know how patients were assigned to groups or the medications they received; only the epidemiologist, who managed randomization and the final data analysis, knew the allocation method. The epidemiologist was not engaged in the prescribing or the assessment of the treatment. 

### Intervention groups and outcomes

The treatment group, patients undergoing PPCI who got standard treatments for acute myocardial infarction, received TSC at a dose of 0.5 mg/kg as an injection, 5 min before primary PCI, and then on days 0 to 3, patients received crocetin tablets orally at a dose of 7.5 mg, 3 times a day. In the placebo group, patients undergoing PPCI who received standard treatments for AMI, got placebo at a dose of 0.5 mg/kg as an injection, 5 min before PCI, and then on days 0 to 3, patients received placebo tablets 3 times a day. 

for the patient at the baseline: Demographic information, including age and sex, underlying diseases such as diabetes mellitus, hypertension, and hyperlipidemia.

The primary outcome was ST-segment resolution ≥ 70% (complete), ST segment resolution was divided into three categories: I. no response to treatment (ST-R levels < 30%), II. partial response to the treatment (30% ≤ ST-R levels < 70%), and III. a complete response to the treatment (ST-R levels ≥ 70%). (Aasa, Henriksson et al. 2010) . A 12-lead ECG was recorded on admission and 60 minutes after PCI. Secondary outcomes were CTIMIFC, prevalences and frequency of arrhythmias until 48 hr after PPCI, evaluation of the patient's echocardiography parameters such as left ventricular ejection fraction(LVEF), left and right ventricular size and pulmonary hypertension until discharge, and major adverse cardiovascular events (MACE) occurring during hospital stays, including death, stroke, ventricular tachycardia, ventricular fibrillation, and heart failure (Akbari, Ghaffari et al. 2020). A cardiologist blinded to the groups did ST-segment resolution, CTIMIFC measurement, and echocardiography.

### Statistical analysis

To determine the sample size using information extracted from the study of Ota et al., (Ota, Nishikawa et al. 2006) the prevalence of Complete ST-R in AMI patients is equal to 0.17, to detect improvement to the level of at least 0.4. The sample size was determined by taking into account the first type error of 0.05 and the power of the test at 80% using G-Power software version 5.1.3. The sample size in each group was 33 patients. 

The data acquired following the clinical phases of the study was initially characterized using central and dispersion metrics. The Shapiro-Wilk test was used to evaluate the normality of quantitative variables. Data present average values ± standard deviation (SD) for continuous variables and proportions for categorical variables. The two independent samples t-test (or Mann-Whitney U test for non-normally distributed variables) was used to compare the quantitative variables among the study groups. The qualitative variables in the study groups were analyzed using the chi-square test (or the Fisher exact test when necessary). The study groups underwent evaluation using paired t-tests or Wilcoxon tests to check for changes. All statistical analyses were performed using SPSS version 25 with a significance threshold of 0.05.

## Results

### Patient’s characteristics

**Figure 1:**
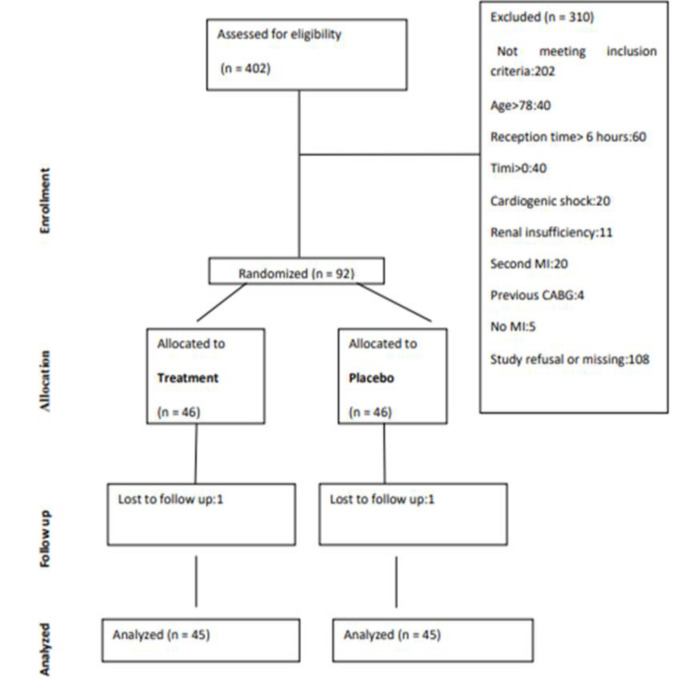
The CONSORT flowchart of the study

A total of 90 patients (TSC group, n = 45 and placebo group, n = 45) were included in the study. The CONSORT flowchart is shown in Figure 1. Patient baseline characteristics are shown in Table 1. There were no significant differences in demographic data, hemodynamic parameters, angiographic data, or past medical history at baseline **(**p>0.05).

### Comparison of one-hour ST-resolution occurrence between the two groups

Regarding the primary outcome, complete ST-segment resolution (equal or more than 70%) occurred in 46.7% of patients in the TSC groups and 20% of patients in the placebo groups. Also, the number of patients with no resolution (equal to 0 to 30%) in the placebo group was significantly more than in the drug group, 28% versus 13% respectively (Table 2) (p=0.018).

**Table 1:** Baseline characteristics of patients

**ST_R**	**TSC No. (%) **	**Placebo** ** No. (%)**	**p value**
Complete	21(46.7%)	9(20%)	0.018^*^
Partial	18(40%)	23(51%)	
No-resolution	6(13%)	13(28%)	

*:Pearson chi-square, ¥:fisher exact test, $: two independent t-test, 1: number 2: percent

**Table 2:** ST-R occurrence comparison between two groups

**Demographics **	**TSC group (n:45)**	**Placebo group (n:45) **	**p Value**
Age	62.14 ± 12.50	58.46 ± 10.68	p**=**0.29^$^
Sex (M/F)	33/12	35/10	p**=**0.62^*^
BMI kg/m^2^	24.02 ± 3.05	24.07 ± 3.55	p**=**0.21^$^
Medical History			
Hypertension no(%)	12(23.7%)	8(19.5%)	p**=**0.39^*^
Diabet Mellitus no(%)	8(18.2%)	5(12.2%)	p**=**0.44^*^
Dyslipidemia no(%)	7(15.9%)	2(4.9%)	p**=**0.158^¥^
Hemodynamic			
Mean atrial pressure	94.7 ± 17.09	97.11 ± 14.77	p**=**0.79^$^
Heart Rate	78.72 ± 11.36	77.33 ± 14.44	p**=**0.094^$^
Angiographic findings			
Infarcted coronary artery			
Left anterior descending artery	26(57.8%)	30(66.7%)	p=0.71^¥^
Right coronary artery no(%)	15(33%)	11(24%)	
Left circumflex coronary artery	4(8.8%)	4(8.8%)	
Percutaneous coronary intervention			
Pre-dilation	25/45	18/45	p**=**0.14^*^
Stent size(long/short)	40/5	42/3	p=0.71^¥^
Post-dilation	18/45	23/45	p**=**0.21^*^
Type of MI			
Anterior no(%)	29(64.4%)	26(57.5%)	p=0.66^¥^
Inferior no(%)	16(35.6%)	19(42.5%)	
Time to PCI(h)	4.12	3.55	p>0.05^*^

*: Pearson’s chi-square

### Comparison of CTIMIFC between the two groups

There was no statistical difference between the TSC and placebo groups in increasing the CTIMIFC parameter (Table 3).

### Comparison of echocardiography parameters between the two groups

There was no statistical difference between TSC and placebo groups in Echo-cardio graphic parameters such as Left Ventricular Ejection Fraction (LVEF) left and right ventricular size, aortic, mitral, and tricuspid valve regurgitation, stenosis, and pulmonary hypertension three days after PPCI (Table 4).

### Comparison of the frequency of arrhythmias between the two groups

Although the frequency of arrhythmias 48 hr after MI was lower in the TSC group compared with the placebo group, the difference was not statistically significant (Table 5).

### Comparison of in-hospital MACE between the two groups

 Major adverse cardiovascular events (MACE) occurred during hospital stays, including death, stroke, ventricular tachycardia, ventricular fibrillation, and heart failure, in the TSC group compared with the placebo group but the difference was not statistically significant (Table 6).

### Comparison of adverse drug reactions between two groups

The adverse drug effects reported by TSC and placebo groups were analyzed and there was no statistical difference between the two groups (Table 7).

**Table 3:** CTIMIFC comparison between two groups

	**TSC group**	**Placebo**	**p value**
CTIMFC	14.2 ± 5.61^*^	13.9 ± 7.13^*^	0.52^α^

α: Mann-Whitney test, *: standard deviation

**Table 4:** Echocardiography parameters comparison between two groups

	**TSC **	**Placebo**	**p value**
LVEF	36.2 ± 11.38^*^	38.12 ± 10.56^*^	0.44^β^
LV enlargement no (%)	0	1(2.7%)	0.46^#^
RV enlargement	0	0	
Pulmonary Hypertension no (%)	0	2(5.8%)	0.22^#^

β: independent sample t-test, #: Fisher's exact test, *: standard deviation

**Table 5:** Frequency of arrhythmias comparison between two groups

	**TSC No. (%)**	**Placebo No (%)**	**p value**
Sinus Tachycardia	22.22%	33.3%	0.23^*^
Atrial Fibrillation (AF)	2(6.6%)	3(8.22%)	0.69^*^
Ventricular Tachycardia (VT)	2(6.6%)	7(15.5%)	0.18^*^
Ventricular Fibrillation (VF)	2(4.4%)	2(4.4%)	1^#^
Premature ventricular contraction (PVC)	7(15.5%)	8(17.7%)	0.77^*^

*: Pearson’s chi-square, #: fisher exact test

**Table 6:** Comparison of in-hospital MACE between two groups

	**TSC no (%)**	**Placebo no (%)**	**p value**
Heart Failure	20(44.4%)	19(42.2%)	0.67^*^
VF	2(4.4%)	2(4.4%)	1^#^
VT	3(6.6%)	7(15.5%)	0.18^*^
Stroke	0	0	
Death	0	0	
Total	22(48.8%)	23(51.1%)	0.83^*^

*: pearson chi-square, #: fisher exact test

**Table 7:** Comparison of drug adverse reactions between two groups

	**TSC**	**Placebo**	**p value**
Headache	12(26%)	15(33%)	P=0.64
Insomnia	4(8.9%)	3(6.9%)	P=1
Drowsiness	0	0	
Agitation	7(15%)	5(11%)	P=0.75
Fatigue	6(13%)	3(7%)	P=0.48
HSR^*^	5(11%)	2(4%)	P=0.45
Nausea	5(11%)	4(8.9%)	P=1
Vomiting	5(11%)	4(8.9%)	P=1
Diarrhea	0	0	
Constipation	4(10%)	3(6%)	P=0.8
Tremor	0	0	
Loss of appetite	0	0	
Increased appetite	0	0	
Injection site reaction	0	0	
Body pain	0	0	
Abdominal pain	0	0	
Flushing	13(30%)	14(33%)	P=1
Palpitation	0	0	

*: hyper sensitivity reactions

## Discussion

Our study findings provided early evidence to suggest that TSC might play a role in reducing reperfusion injury in AMI. Specifically, we found that the administration of TSC just before PPCI was associated with a higher occurrence of complete ST_R (≥70%) in the drug group (46.7%) than in patients who received placebo (20%). TSC could not affect CTIMIFC, echocardiography parameters, frequency of arrhythmias, and in-hospital MACE in the treatment versus placebo groups. 

The reason for evaluating the effect of TSC as an antioxidant and cardioprotective agent in individuals experiencing AMI stems from experimental findings that highlight the significant impact of oxidative stress and ROS in reperfusion injury (Braunwald and Kloner 1985, Sharma, Bell et al. 2012, Fröhlich, Meier et al. 2013, Bainey and Armstrong 2014, Granger and Kvietys 2015, Zhou, Chuang et al. 2015, Caccioppo, Franchin et al. 2019). However, data from *in vivo* and *in vitro* research mentioned below, support the hypothesis that TSC as an antioxidant, anti-hypoxia, and cardioprotective agent appears to have beneficial effects in reducing reperfusion injury. Recent studies have also highlighted the significance of ST resolution as a valuable marker of myocardial reperfusion in the microvascular system and have been shown to correlate with patient survival (Galiuto, Garramone et al. 2008, Jia, Nie et al. 2016, Matin, Ghaffari et al. 2020). The resolution of the ST segment is crucial in assessing myocardial injury and reperfusion damage in STEMI patients, enhanced resolution is associated with a better prognosis, while incomplete or worsening resolution correlates with larger infarct sizes and a higher risk of negative outcomes (Schroder 2004, Reinstadler, Baum et al. 2015). In summary, ST resolution is a marker of myocardial reperfusion at the microvascular level and significantly correlates with myocardial reperfusion (de Lemos and Braunwald 2001).

Chang et al study has demonstrated that TSC exerts a cardioprotective effect against myocardial infarction/reperfusion (MI/R) injury and TSC attenuates the oxidative stress, apoptosis, and mitochondrial dysfunction induced by MI/R through the SIRT3/FOXO3a/SOD2 signaling pathway. These results suggested that TSC may serve as a potential therapeutic agent for the treatment of patients with myocardial infarction, given its ability to mitigate the harmful effects of MI/R injury on the myocardium (Chang, Chen et al. 2019). Deng et al results suggest that 0.14 mg/kg TSC given under the bolus–infusion–bolus regimen provides neuroprotection in obese mice. This effect may involve reduced oxidative stress, inflammation, MMP-9, and activity in the ischemic brain tissues (Deng, Xiong et al. 2015).

Aminifard et al found that TSC could attenuate the cytotoxic effects of myoglobin on HEK-2 cells by inhibiting the induction of apoptotic and oxidative responses (Aminifard, Mehri et al. 2023). Additionally, TSC has been reported to protect against contrast-induced cytotoxicity in HEK-293 cells by decreasing intracellular ROS generation and enhancing the activity of endogenous antioxidant enzymes (Rajabian, Mehri et al. 2023). Stennett et al study on mice with hemorrhagic shock showed that TSC could scavenge free radicals *in vitro* (Stennett, Murray et al. 2007).

One study on patients with COVID-19 demonstrated that TSC is safe and can improve oxygenation in case of hypoxemia. (Streinu-Cercel, Săndulescu et al. 2021). Another study involving 59 patients with glioblastoma multiform (GBM) suggests that adding TSC during RT is beneficial for treating GBM because it combats hypoxia in tumor tissue (Gainer, Sheehan et al. 2017).

TIMIFC (a crucial determinant of myocardial perfusion) is a reliable indicator of epicardial coronary flow, (Matin, Ghaffari et al. 2020) and is widely used to compare the effectiveness of thrombolytics (Gibson, Cannon et al. 1996). TSC could not increase TIMIFC because the known mechanisms of this compound, do not include any effect on epicardial coronary flow.

In our study, there were no differences between echocardiographic parameters such as LVEF in two groups three days after MI. This lack of response may be attributed to the acute phase of MI, which often presents with stunned or hibernating myocardium, affecting the reliability of echocardiographic assessments due to these transient changes (Flachskampf, Schmid et al. 2011). To obtain a more accurate evaluation, it is suggested that echocardiography be carried out in the cardiac recovery phase at least a few weeks after MI. Further studies or echocardiography with longer follow-ups are needed to evaluate these parameters precisely. 

Arrhythmias frequency and prevalence, especially ventricular arrhythmia, are crucial prognostic factors. Studies show that frequent ventricular arrhythmias increase the risk of death in post-heart attack patients (Maggioni, Zuanetti et al. 1993). The frequency of arrhythmias decreased but it was not significant between the two groups. Post-MI arrhythmia is caused by factors such as autonomic dysfunction, electrolyte imbalances, hypoxia, and tissue damage. It leads to increased myocardium automaticity, altered refractoriness, and re-entry circuits in damaged tissues (Bhar-Amato, Davies et al. 2017). As mentioned above, the pathophysiology of cardiac arrhythmia after MI is complex and one of these pathways is increased oxidative stress.

Treatment of reperfusion injury in STEMI patients must begin before reperfusion, ideally during first medical care to angioplasty, as most damage occurs in the first minute of reflow (Morel, Perret et al. 2012). Because of this documentation, we used TSC immediately before PPCI.

 Animal toxicology studies have shown that high levels of TSC are well tolerated and a phase I clinical trial has shown that TSC is safe in humans (Gainer 2008). The data from our study also confirm that TSC did not significantly increase any of the possible adverse effects compared to placebo.

Our study had limitations such as a small sample size and a short follow-up period. We just evaluated the patients in the short term, further evaluation is recommended. Additional research is necessary to validate and build upon these findings. Nonetheless, our results suggest TSC could be a beneficial treatment for reducing reperfusion injury adverse effects in AMI patients. These data are preliminary and require confirmation in a larger clinical trial.

The administration of TSC in patients with ST-elevation myocardial infarction (STEMI) undergoing primary percutaneous coronary intervention (PPCI) significantly improved ST-segment resolution, suggesting a reduction in reperfusion injury. While this study positively impacted the primary outcome, TSC did not considerably affect secondary outcomes, including corrected thrombolysis in myocardial infarction (CTIMI) flow grade, echocardiographic parameters, or the incidence of supraventricular and ventricular arrhythmias. These findings indicate that TSC may be beneficial in enhancing myocardial reperfusion, but further research with larger cohorts and extended follow-up is necessary to fully understand its potential and mechanisms of action in MI treatment.

## Abbreviations

AMI: Acute myocardial infarction; CAD: Coronary artery disease; CTIMIFC: Corrected thrombolysis in myocardial infarction frame count; LVEF: Left ventricular ejection fraction; LV: Left ventricular; MRI: Myocardial reperfusion injury; PPCI: Primary percutaneous coronary intervention; ROS: Reactive oxygen species; STEMI: ST-elevation myocardial infarction; TSC: Trans sodium crocetinate.
